# 

**Published:** 1872-05-15

**Authors:** 


Money Receipts to May 30th, 1872.—Dr. Newland, $6.00; C. J. Miller, $3.00; James Brooks, $6.00; Henry Young, $3.00; J. P. Mathews, $3.00; J. C. Spohn, $3.00; Joseph Fortier, $2.00; T. D. Fisher, $3.00.

